# On the prevalence of racial discrimination in the United States

**DOI:** 10.1371/journal.pone.0210698

**Published:** 2019-01-10

**Authors:** Randy T. Lee, Amanda D. Perez, C. Malik Boykin, Rodolfo Mendoza-Denton

**Affiliations:** 1 Department of Psychology, Cornell University, Ithaca, New York, United States of America; 2 Department of Psychology, University of California, Berkeley, Berkeley, California, United States of America; 3 Department of Cognitive, Linguistic, and Psychological Sciences, Brown University, Providence, Rhode Island, United States of America; Iranian Institute for Health Sciences Research, ISLAMIC REPUBLIC OF IRAN

## Abstract

Boutwell, Nedelec, Winegard, Shackelford, Beaver, Vaughn, Barnes, & Wright (2017) published an article in this journal that interprets data from the Add Health dataset as showing that only one-quarter of individuals in the United States experience discrimination. In Study 1, we attempted to replicate Boutwell et al.’s findings using a more direct measure of discrimination. Using data from the Pew Research Center, we examined a large sample of American respondents (*N* = 3,716) and explored the prevalence of discrimination experiences among various racial groups. Our findings stand in contrast to Boutwell et al.’s estimates, revealing that between 50% and 75% of Black, Hispanic, and Asian respondents (depending on the group and analytic approach) reported discriminatory treatment. In Study 2, we explored whether question framing affected how participants responded to Boutwell’s question about experiencing less respect and courtesy. Regardless of question framing, non-White participants reported more experiences than White participants. Further, there was an interaction of participant race and question framing such that when participants were asked about experiences of less respect or courtesy broadly, there were no differences between non-White participants and White participants, but when they were asked about experiences that were specifically race-based, non-White participants reported more experiences than White participants. The current research provides a counterweight to the claim that discrimination is not a prevalent feature of the lives of minority groups and the serious implications this claim poses for research and public policy.

## Introduction

A recent article by Boutwell, Nedelec, Winegard, Shackelford, Beaver, Vaughn, Barnes, & Wright [1] published in PLOS ONE on the prevalence of discrimination across racial groups has received a fair amount of attention [1–4]. Their findings appear to show that only a small minority of individuals across all racial groups experience discrimination, and comments on social media reflect an acceptance of the authors’ conclusions: “What a shock, it's almost as if America is an egalitarian society that spent over 200 years fighting for equal protection under the law,” “most of what comes from the political left, when exposed to reality, scrutiny and fact turns out to be myth,” and “discrimination is not the juggernaut it is constantly cracked up to be.” Even scientists and science writers [5–7] have responded publicly to the findings. The response to Boutwell et al. underscores the influence that researchers can have on narratives around sensitive issues, and the responsibility that researchers have towards the conclusions they draw from data, especially for work that is sensitive and polarizing.

To recap, Boutwell and colleagues examined the National Representative Longitudinal Study of Adolescent to Adult Health (Add Health) [8] and interpret their data as revealing that only one-quarter of all participants (25.20%), regardless of race, faced discrimination. Between racial groups, they found prevalence rates of 23.53% for Whites, 31.88% for Blacks, 27.15% for Hispanics, 11.61% for American Indians, 18.72% for Asians, and 26.99% for mixed-race individuals. Moreover, they write, “of those reporting having experienced discrimination, the majority suggested that unique and perhaps situationally specific factors other than race, gender, sexual orientation, and age were the cause(s) of discrimination.”

To measure discrimination, Boutwell and colleagues used the following single item question from the Add Health Wave IV in-home interview from Add Health: “*In your day to day life*, *how often do you feel you have been treated with less respect or courtesy than other people*?” (0 = *never*, 1 = *rarely*, 2 = *sometimes*, and 3 = *often)*. The researchers dichotomized the responses to this item such that responses of *never* and *rarely* were collapsed as *No*, while *sometimes* and *often* were collapsed as *Yes*. Individuals who responded with *sometimes* or *often* in the Add Health survey were subsequently asked, “What do you think was the main reason for these experiences?” and told to pick one reason: (1) your ancestry or national origin; (2) your gender, (3) your race; (4) your age; (5) your religion; (6) your height or weight; (7) your shade of skin color; (8) your sexual orientation; (9) your education or income; (10) a physical disability; or (11) other. Boutwell et al. collapsed ancestry or national origin, race, and shade of skin color to one category “Race/Ancestry/Skin color.” Responses to these items were matched to a Wave I in-school interview question that captured racial and ethnic demographics of the respondent, which was administered when participants were 12 to 18 years old. Over 99% of participants reported speaking English during Wave I, though 2% of the interviews were conducted in Spanish. In the subsequent Waves, less than 0.2% of interviews were conducted in Spanish. By Wave IV, the respondents were adults aged 24 to 32 years old. For more information about the Add Health dataset, see https://www.cpc.unc.edu/projects/addhealth/design.

### Respect, discrimination, and framing effects

As noted, Boutwell and colleagues used “*In your day to day life*, *how often do you feel you have been treated with less respect or courtesy than other people*?” as their index of discrimination. This question, although having a different response scale, is a composite of two questions from the nine-item Everyday Discrimination Scale (EDS), which was constructed to measure “chronic, routine, and relatively minor experiences of *unfair treatment*” [9, 10]. Other items in the scale deal with receiving poorer service than others in restaurants and people acting as if you are not smart. We believe that researchers who want to measure everyday experiences of unfair treatment due to race or another status-based characteristic should use the full scale or (at the very least) multiple items from it and specify the status-based characteristic being examined. Critically, researchers have found that questions that ask about unfair treatment and racial/ethnic discrimination are two “qualitatively different phenomena” (e.g., [11,12]).

We argue that the variable used by Boutwell and colleagues to examine discrimination should be more appropriately referred to as respect, rather than discrimination. This distinction is critical. One can be denied a loan, turned down from a job, or provided unequal opportunity on the basis of one’s background or identity—yet done in a respectful and courteous fashion. Consistent with this idea, Saguy, Tausch, Dovidio, & Pratto [13] experimentally found that even after positive, polite, and amicable interactions, individuals were still discriminated against by the advantaged out-group in a resource allocation task.

The distinction between respect and discriminatory treatment may be one clue to helping reconcile Boutwell et al.’s findings with a wealth of prior literature on the prevalence of discrimination. Using the MacArthur Foundation Midlife Development in the United States (MIDUS) survey, Kessler, Mickelson, and Williams [14] found that 60.9% of all respondents experienced some form (*rarely*, *sometimes*, *often*) of at least one of the nine EDS items and 33.5% of all individuals experienced an instance of *major* discrimination in their lifetime in a national sample of Americans aged 25–74. The EDS included items related to being treated as if not smart, inferior, or dishonest, whereas major discrimination included items like being denied a bank loan, hassled by the police, or forced to leave a neighborhood due to discrimination. We note that the MIDUS used scales consisting of multiple items for both major and daily experiences, rather than single-item questions. Collapsing *often* and *sometimes* for the EDS items (like Boutwell and colleagues did with the single Add Health measure) in the MIDUS dataset results in a 23.7% day to day discrimination prevalence for Whites and 71.3% for Blacks (see Table 3 in Kessler, Mickelson, & Williams [14]). For major lifetime discrimination, 30.9% of Whites compared to 48.9% of Blacks reported experiencing major discrimination (see Table 1 in Kessler, Mickelson, & Williams [14]). Highlighting the dramatic differences between the groups, 44.4% of Whites reported never experiencing day to day discrimination compared to 8.8% of Blacks. Similarly, the 2002–2003 Collaborative Psychiatric Epidemiology Studies (CPES) included all nine items from the EDS in their study of Black, Asian, and Hispanic individuals. Like the Add Health survey, respondents were able to choose the reason for their mistreatment (e.g., ancestry, gender, age, height or weight) after responding to each of the nine questions. Using this dataset, Chou, Asnaani, and Hofmann [15] found that 58% of Blacks, 39% of Asians, and 35% of Hispanics reported racial reasons for their mistreatment.

In a 2017 nationally representative study on prevalence of institutional discrimination in America, NPR, the Robert Wood Johnson Foundation, and the Harvard T.H. Chan School of Public Health found that 60% of Blacks (49% in urban areas and 67% in suburban areas) reported that they or a family member had been unfairly treated or stopped by a police officer due to race, compared to 27% of Hispanics, 13% of Asians, and 6% of Whites. The 2017 study also found that 57% of Blacks reported discrepancies in earning equal pay or promotions, compared to 32% of Hispanics, 31% of Native Americans, 25% of Asian Americans, and 13% of Whites [16]. We note institutional (also known as structural) discrimination is not at all captured by the respect variable used by Boutwell and colleagues. Institutional discrimination refers to the disparities that systematically favor certain groups, and scholars have noted that in order to understand racial disparities across multiple domains (e.g., housing, schooling, employment, health, justice), these disparities have to be seen as “reciprocally related and comprise an integrated system” [17].

These findings highlight how there can be a disconnect between the concepts of perceiving that oneself was treated with less respect, perceiving that one has experienced discrimination, and being a target of discrimination. Taken together, the findings suggest that the overall prevalence rate of racial discrimination is likely over 25% in the United States, and that the prevalence rate should vary significantly by racial groups.

#### Does question framing matter?

Central to the issue of discrepant prevalence rates between Boutwell et al. and previous findings is framing. According to Entman [18], framing is done to “promote a particular problem definition, causal interpretation, moral evaluation, and/or treatment recommendation.” Frames work by drawing attention to a particular aspect of information, which makes the piece of information more noticeable, meaningful, or memorable [18]. The same issue framed in different ways has been found to reliably produce shifts in responses [19]. Indeed, Brown [20] found differences in the prevalence rate of self-perceived racial and ethnic discrimination for Blacks based on framing. Depending on whether he used one explicitly framed question about experiencing unfair treatment due to race and ethnicity or six unfair treatment two-stage questions (e.g., Stage 1: Have you been treated unfairly? Stage 2: What was the reason?), he found a 67% prevalence rate (explicit) compared to a 50% prevalence rate (two-stage). In sum, unfair treatment due to race-based discrimination was 1.34 times more likely to be reported if the question framing was explicit and was direct. Boutwell et al’s item, in the absence of explicit framing about discrimination or a status-based characteristic (e.g., race, gender, age), may have led to under-reporting of participants’ experiences.

### The current research

In Study 1, we present analyses that parallel and attempt to replicate the findings of Boutwell et al. using a nationally representative dataset from the Pew Research Center, which has a question that explicitly and more directly measures experiences of racial discrimination. In Study 2, we experimentally manipulate framing to see whether it can shift responses to a question on being treated with less respect and courtesy. We hypothesize that a broad framing of experiencing less respect leads to a minimization of racial differences, but as the framing becomes more specific and draws attention to race and ethnicity, differences will be revealed.

Study 1 was determined to by the UC Berkeley’s Committee for the Protection of Human Subjects (CPHS) Institutional Review Board (IRB) to be secondary data analyses, thus not needing IRB approval. Study 2 was reviewed and approved by UC Berkeley's CPHS IRB under protocol 2018-04-10951. In Study 2, participants provided digital consent by typing their MTurk ID.

## Study 1

### Method

#### Data

**Pew Research Center Data.** We utilized the Pew Research Center’s 2016 Racial Attitudes in America Survey dataset [21], which has a question that directly asks about personal experiences with discrimination. The data were collected using telephone interviews conducted between February 29 to May 8, 2016, among a nationally representative sample of adults, 18 years of age or older, living in all 50 U.S. states and the District of Columbia. The interviews were conducted in both English and Spanish, with most (74.1%) interviewed on cell phones. The Hispanic sample in the dataset were predominantly native born and English speaking.

Our sample for analyses had a total of 3,631 participants (M_age_ = 49.62). The gender composition consisted of 1,903 male (52.41%) and 1,728 female (47.59%), and the racial composition consisted of 2,094 White (57.67%), 1,077 Black (29.66%), 129 Asian (3.55%), and 331 Hispanic (9.12%). Respondents were only able to select one racial identity. See Table 1 for full breakdown of race by response.

**Weights.** We used probability weights to adjust for the sampling method of the survey. Weighting is typically used in survey data analysis to adjust for effects of the sample design and to compensate for potentially biased sampling. The Pew Research Center’s 2016 Racial Attitudes in America Survey dataset [21] states that their weighting variable was created to account for “the disproportionately-stratified samples, the overlapping landline and cell sample frames and household composition, the oversampling of African-Americans through callback interviews, and differential non-response associated with sample demographics.” For more information about the Pew Research Center weighting and methodology, see http://www.pewresearch.org/methodology/u-s-survey-research/our-survey-methodology-in-detail/.

We used the *svydesign* and *svyglm* functions from the *survey* package in R to conduct our analyses with the appropriate weights, using the *weight* variable in the dataset that the Pew Research Center created and provided.

**Racial Discrimination Measure.** We used the item, “*Have you ever personally experienced discrimination or been treated unfairly because of your race or ethnicity*, *or not*?” [If respondents answered *Yes*: “*And would you say this is something you experience regularly*, *or is this something you experience from time to time*, *but not regularly*?”] (1 = *Yes*, *regularly*, 2 = *Yes*, *from time to time*, 3 = *Yes*, *but only one time/rarely*, 4 = *No*, and 9 = *Don’t know/Refused*). For the analyses, individuals who responded with *Don’t Know/Refused* were removed and the responses were reverse coded, such that higher numbers reflected more racial discrimination experiences.

#### Analytic Plan

We ran three different models with the dependent variable of racial discrimination in order to parallel the analytic plan of Boutwell and colleagues, who ran models using categorical versions and dichotomous versions of their measure. The first model treats the racial discrimination variable as a Likert, continuous variable, while the second and third models treats the racial discrimination variable as a *No* and *Yes* dichotomous variable. We dichotomized the variable two ways: the first of these (Model 2) was conducted such that 0 reflects *No* experience and the rest (1, 2, 3) reflected at least one instance of racial discrimination. The second dichotomization (Model 3) was conducted with 0 and 1 reflecting *No* experience (collapsed and coded as zero) and the rest (2, 3) reflecting experiencing discrimination more consistently than not (collapsed and coded as 1). The model that most closely corresponds to Boutwell et al. is Model 3.

## Results

### Prevalence of discrimination

43.50% of all individuals in the Pew Research Center dataset reported experiencing discrimination from *time to time* or *regularly*. This is a substantial difference compared to the 25.20% reported by Boutwell and colleagues. When comparing the experiences of discrimination of minorities (Black, Hispanic, and Asian) and Whites, we also find a large contrast in our results. In our most conservative estimates, we find 63.10% of minorities experience racial discrimination compared to 29.61% of Whites. By contrast, Boutwell et al. report estimates of 28.74% of less respect or courtesy for minorities and 23.53% for Whites. Group level prevalence rates paint an even larger difference from Boutwell et al.’s estimates. In our conservative estimate, 69.45% of Blacks experience discrimination from *time to time* or *regularly* in comparison to the 31.88% reported by Boutwell and colleagues. Other differences are the prevalence rates found for Asians (56.59% in our study compared to 18.72% in the Boutwell et al.) and for Hispanics (45.01% in our study compared to 27.15% in Boutwell et al.).

Table 1 includes the total breakdown of frequencies in our sample, while Tables 2 and 3 has dichotomized responses. Tables 2 and 3 differ at the point where the discrimination variable is dichotomized and respectively complement Model 2 and Model 3 in our analyses. For ease of comparison, these tables are formatted similarly to Table 1 in Boutwell et al. [1].

**Table 1:** Breakdown of Pew Center data experiences of reported discrimination.

	*Discrimination Experience*					
	Don’t know or Refused	No *(0)*	Yes, but only one time/rarely *(1)*	Yes, from time to time *(2)*	Yes, regularly *(3)*	*M* and *SD*
White (*n* = 2,094)	0.67%	64.85%	4.87%	25.79%	3.82%	*M* = 0.68
	14	1,358	102	540	80	*SD* = 0.98
Black (*n* = 1,077)	1.30%	25.07%	4.18%	58.22%	11.23%	*M* = 1.56
	14	270	45	627	121	*SD* = 0.99
Asian (*n* = 129)	0.00%	40.31%	3.10%	45.74%	10.85%	*M* = 1.27
	0	52	4	59	14	*SD* = 1.11
Hispanic (*n* = 331)	0.00%	50.45%	4.53%	36.86%	8.16%	*M* = 1.03
	0	167	15	122	27	*SD* = 1.10
American Indian (*n* = 49)	2.04%	44.90%	4.08%	38.78%	10.20%	*M* = 1.15
	1	22	2	19	5	*SD* = 1.13
Pacific Islander (*n* = 7)	0.00%	28.57%	0.00%	57.14%	14.29%	*M* = 1.57
	0	2	0	4	1	*SD* = 1.33
Other (*n* = 9)	0.00%	33.33%	0.00%	55.56%	11.11%	*M* = 1.44
	0	3	0	5	1	*SD* = 1.13
Don’t know (*n* = 20)	5.00%	35.00%	10.00%	40.00%	10.00%	*M* = 1.26
	1	7	2	8	2	*SD* = 1.10
Total (*N* = 3,716)	0.01%	50.62%	4.57%	37.24%	6.75%	*M* = 1.00
	30	1,881	170	1,384	251	*SD* = 1.08

Note: For each category, the first row contains the percentage and the second row contains the number of responses. Individuals who identified as Arab and Middle Eastern were categorized as White by the Pew Research Center. Those who identified as Mexican, Puerto Rican, and Cuban were categorized as Hispanic by the Pew Research Center. There are 23 responses that are unknown or missing.

**Table 2:** Pew Research Center data dichotomized. *No* reflects individuals who reported no experiences of discrimination, and *Yes* reflects reports of *One Time/Rarely*, *Time to time*, *and Regular* experiences of discrimination.

	*Dichotomized Responses*		*M* and *SD*
	No *(0)*	Yes *(1)*	
White (*n* = 2,094)	64.85%	34.48%	*M* = 0.35
	1,358	722	*SD* = 0.48
Black (*n* = 1,077)	25.07%	73.62%	*M* = 0.75
	270	793	*SD* = 0.44
Hispanic (*n* = 331)	50.45%	49.55%	*M* = 0.50
	167	164	*SD* = 0.50
Asian (*n* = 129)	40.31%	59.69%	*M* = 0.60
	52	77	*SD* = 0.49
American Indian (*n* = 49)	44.90%	53.06%	*M =* 0.54
	22	26	*SD =* 0.50
Pacific Islander (*n* = 7)	28.57%	71.43%	*M =* 0.71
	2	5	*SD =* 0.49
Other (*n* = 9)	33.33%	66.67%	*M =* 0.67
	3	6	*SD =* 0.50
Don’t know (*n* = 20)	35.00%	60.00%	*M =* 0.63
	7	12	*SD =* 0.50
Total (*N* = 3,716)	50.62%	48.57%	*M =* 0.49
	1,881	1,805	*SD =* 0.50

Note: For each category, the first row contains the percentage and the second row contains the number of responses. Individuals who identified as Arab and Middle Eastern were categorized as White by the Pew Research Center. Those who identified as Mexican, Puerto Rican, and Cuban were categorized as Hispanic by the Pew Research Center. There are 23 responses that are unknown or missing.

**Table 3:** Pew Research Center data dichotomized. *No* reflects individuals who reported no experiences or one time/rare experiences of discrimination, and *Yes* reflects responses of experiencing discrimination from time to time and regularly.

	*Dichotomized Responses*		*M* and *SD*
	No *(0)*	Yes *(1)*	
White (*n* = 2,094)	69.72%	29.61%	*M* = 0.30
	1,460	620	*SD* = 0.46
Black (*n* = 1,077)	29.25%	69.45%	*M* = 0.70
	315	748	*SD* = 0.46
Hispanic (*n* = 331)	54.98%	45.01%	*M* = 0.45
	182	149	*SD* = 0.50
Asian (*n* = 129)	43.41%	56.59%	*M* = 0.57
	56	73	*SD* = 0.50
American Indian (*n* = 49)	48.98%	48.98%	*M =* 0.50
	24	24	*SD =* 0.51
Pacific Islander (*n* = 7)	28.57%	71.43%	*M =* 0.71
	2	5	*SD =* 0.49
Other (*n* = 9)	33.33%	66.67%	*M =* 0.67
	3	6	*SD =* 0.50
Don’t know (*n* = 20)	45.00%	50.00%	*M =* 0.53
	9	10	*SD =* 0.51
Total (*N* = 3,716)	55.19%	43.50%	*M =* 0.44
	2,051	1,635	*SD =* 0.50

Note: For each category, the first row contains the percentage and the second row contains the number of responses. Individuals who identified as Arab and Middle Eastern were categorized as White by the Pew Research Center. Those who identified as Mexican, Puerto Rican, and Cuban were categorized as Hispanic by the Pew Research Center. There are 23 responses that are unknown or missing.

#### Differences in discrimination experiences between groups

Due to sample size and power concerns, we only examined White, Black, Hispanic, and Asian respondents for between group comparison analyses. All racial minority groups (Black, Hispanic, and Asian) reported facing more racial discrimination in comparison to Whites, with Blacks reporting the most among the groups analyzed. Among minorities, Hispanics reported facing less discrimination than Blacks. Depending on how we dichotomized the variables, we found slight differences in experiences: Asians reported facing more discrimination than Hispanics in both Model 1 and Model 3, but there were no differences in Model 2, and Asians reported facing less discrimination than Blacks in Model 2, but there were no differences found in Model 1 and Model 3.

In the next subsections, we dive into each model that we highlighted previously.

#### Model 1: Discrimination using the full Likert range

We found that all racial minority groups (Black, Hispanic, and Asian) reported facing more racial discrimination than Whites, *R*^*2*^ = 0.13, *F*(3, 3599) = 184.7, *p* < .001. Follow-up analyses were conducted to see whether there were differences among the minority groups in experiences of racial discrimination. Hispanics reported experiencing less discrimination than Blacks (*d* = 0.53, *b* = -0.51, *SE* = 0.08, *t* = -6.29, *p* < .001, 95% CI [-0.67, -0.35]) and Asians reported facing more discrimination than Hispanics (*d* = 0.22, *b* = 0.31, SE = 0.15, *t* = 2.12, *p* = .03, 95% CI [0.02, 0.59]). No differences were found between Blacks and Asians in experiences of racial discrimination (*d* = 0.26, *b* = -0.20, *SE* = 0.13, *t* = -1.49, *p* = .14, 95% CI [-0.46, 0.06]). See Table 4 for a summary of racial minority group differences in comparison to Whites.

**Table 4:** Racial discrimination experiences compared to Whites.

	*d*	*b*	*SE*	*t*	*p*	95% CI
Black	0.89	0.89	0.05	18.05	< .001***	[0.79, 0.98]
Hispanic	0.35	0.38	0.07	5.11	< .001***	[0.23, 0.52]
Asian	0.56	0.69	0.13	5.25	< .001***	[0.43, 0.94]

Note: Table 4 corresponds with Model 1, which examined experiences as a Likert scale.

***p < .001,

**p < .01,

*p < .05, p < .10.

#### Model 2: Discrimination as dichotomous split, with *No* vs. *Yes*, *rarely*, *Yes*, *time to time*, and *Yes*, *regularly*

Identical to Model 1, we found that all racial minority groups (Black, Hispanic, and Asian) reported facing more racial discrimination than Whites, with Blacks by far experiencing the largest difference in experiences, *R*^*2*^ = 0.13, *F*(3, 3599) = 173.2, *p* < .001. We ran follow-up analyses to see whether there were differences among the minority groups in experiences of racial discrimination as a dichotomized response. We found that Hispanics faced less discrimination than Blacks (*d* = 0.55, *b* = -0.93, *SE* = 0.16, *t* = -5.69, *p* < .001, 95% CI [-1.25, -0.61]) and a marginal difference between Asians and Blacks, with Asians reporting less experiences of discrimination than Blacks (*d* = 0.30, *b* = -0.49, *SE* = 0.26, *t =* -1.92, *p* = .0549, 95% CI [-1.00, 0.01]). There were no differences between Hispanics and Asians in experiences of racial discrimination (*d* = 0.20, *b* = 0.44, *SE* = 0.28, *t* = 1.59, *p* = .11, 95% CI [-0.10, 0.98]). See Table 5 for a summary of racial minority group experiences of discrimination in comparison to Whites.

**Table 5:** Racial discrimination as dichotomous responses, with No reflecting individuals who reported no experiences, and Yes reflecting One Time/Rarely, Time to time, and Regular experiences of discrimination.

	*d*	*b*	*SE*	*t*	*p*	95% CI
Black	0.92	1.68	0.11	15.40	< .001***	[1.47, 1.89]
Hispanic	0.31	0.75	0.15	5.05	< .001***	[0.46, 1.04]
Asian	0.52	1.19	0.25	4.81	< .001***	[0.70, 1.67]

Note: Table 6 corresponds with Model 3, which examined experiences as dichotomized.

***p < .001,

**p < .01,

*p < .05, p < .10.

#### Model 3: Discrimination as dichotomous split, with *No* and *Yes*, *rarely* vs. *Yes*, *time to time* and *Yes*, *regularly*

Identical to both previous models, we found that all racial minority groups (Black, Hispanic, and Asian) reported facing more racial discrimination than Whites when analyzing the variable as a dichotomized response, *R*^*2*^ = 0.13, *F*(3, 3599) = 183.5, *p* < .001. Again, Blacks experienced the most amount of discrimination in comparison to Whites. We ran follow-up analyses to see whether there were differences among the minority groups in experiences of racial discrimination as a dichotomized response. We found that Hispanics reported facing less discrimination than Blacks (*d* = 0.54, *b* = -0.98, *SE* = 0.16, *t* = -6.07, *p* < .001, 95% CI [-1.30, -0.67]) and Asians reported facing more discrimination than Hispanics (*d* = 0.23, *b* = 0.63, *SE* = 0.27, *t* = 2.33, *p* = .02, 95% CI [0.10, 1.17]). There were no differences between Blacks and Asians (*d* = 0.28, *b* = -0.35, *SE* = 0.25, *t* = -1.37, *p* = .17, 95% CI [-0.84, 0.15]). See Table 6 for a summary of racial minority group experiences in comparison to Whites.

**Table 6:** Racial discrimination as dichotomous responses, with No reflecting individuals who reported No experiences and One Time/Rarely, and Yes reflecting individuals who reported Time to time, and Regular experiences of discrimination.

	*d*	*b*	*SE*	*t*	*p*	95% CI
Black	0.89	1.72	0.11	16.06	< .001***	[1.51, 1.93]
Hispanic	0.33	0.74	0.15	4.92	< .001***	[0.45, 1.03]
Asian	0.56	1.38	0.25	5.60	< .001***	[0.89, 1.86]

Note: Table 6 corresponds with Model 3, which examined experiences as dichotomized.

***p < .001,

**p < .01,

*p < .05, p < .10.

### Discussion

Like Boutwell et al., we were not able to analyze all racial groups in our sample. We note that there is much discussion whether it is appropriate or valid to dichotomize items that were originally continuous variables (e.g., [22,23]). For the purposes of our analyses, we nevertheless felt that it was appropriate to conduct parallel analyses to Boutwell and colleagues. In our most conservative analyses (Model 3), we find that between 45% and 70% of Black, Hispanic, and Asian respondents report experiencing racial discriminatory treatment compared to 30% of Whites. Collapsing across racial groups, we find that around 63% of minorities (Black, Hispanic, and Asian) experience discrimination compared to 30% of Whites. In contrast, Boutwell and colleagues found a prevalence rate of 29% for minorities and 24% for Whites. Overall, we find that nearly 43% of all individuals experience discrimination, a substantial difference compared to the 25% reported by Boutwell and colleagues. However, a remaining question is why there are such discrepant prevalence rates between our findings and those of Boutwell and colleagues. One possibility is sampling error; however, given the relatively robust sample sizes, we believe sampling error alone is unlikely to account for the drastic differences. Additionally, the item we used to measure discrimination directly asked participants whether they had experienced discrimination or been treated unfairly due to race or ethnicity. As noted earlier, framing questions as being explicitly about discrimination can change how participants respond and this may account for the discrepancy between our findings and Boutwell et al.’s. We address this possibility directly in Study 2 by experimentally manipulating the framing of the exact question used by Boutwell and colleagues.

## Study 2

### Method

#### Participants and design

394 participants (M_age_ = 35.38) from Amazon Mechanical Turk participated in a study on experiences. Participants were required to be native English speakers living in the United States. The gender composition consisted of 221 Male (56.1%), 170 Female (43.1%), 2 Non-Binary, and 1 unknown, and the racial composition consisted of 302 White (76.6%), 31 Black (7.9%), 30 Asian (7.6%), 16 Hispanic (4.1%), 11 two or more (2.8%), 2 other, 1 American Indian/Alaskan, and 1 Native Hawaiian and/or Other Pacific Islander. In the subsequent analyses, we collapsed across race and compared White (*n* = 302) and non-White (*n* = 92) participants due to small sample sizes within the racial minorities collected.

All participants were randomly assigned to one of three conditions and completed a single item about experiencing less respect or courtesy based on either (1) Less Respect: “*In your day to day life*, *how often do you feel you have been treated with less respect or courtesy than other people*?”; (2) Any Type of Discrimination: “*In your day to day life*, *how often do you feel you have been treated with less respect or courtesy than other people because of your race*, *ethnicity*, *gender*, *disabilities*, *sexual orientation*, *or age*?”; or (3) Race-Based Discrimination: “*In your day to day life*, *how often do you feel you have been treated with less respect or courtesy than other people because of your race or ethnicity*?” We note that the “Less Respect” framing condition is identical to the question used by Boutwell and colleagues. Like the Add Health response scale and the data analyzed by Boutwell and colleagues, participants rated their responses on a 0–3 scale, with 0 = *never*, 1 = *rarely*, 2 = *sometimes*, and 3 = *often*.

### Results

For ease of readability, we refer to the outcome variable as simply "experiences" and we will distinguish between each condition in the relevant analyses. To distinguish each condition, we will use “Less Respect”, “Any Type of Discrimination”, and “Race-Based Discrimination”. See Table 7 for a breakdown of responses by each question framing condition.

**Table 7:** Breakdown of experiences of Study 2 by question framing condition.

	Never *(0)*	Rarely *(1)*	Sometimes *(2)*	Often *(3)*			*M* and *SD*
***Because of Less Respect***							
White (*n* = 106)	11.32%	54.72%	29.25%	4.72%			*M* = 2.27
	12	58	31	5			*SD* = 0.72
non-White (*n* = 31)	12.90%	58.06%	19.35%	9.68%			*M* = 2.26
	4	18	6	3			*SD* = 0.82
***Because of Any Type of Discrimination***							
White (*n* = 96)	26.80%	41.24%	25.77%	5.15%			*M* = 2.09
	26	40	25	5			*SD* = 0.86
non-White (*n* = 30)	9.68%	38.71%	45.16%	3.23%			*M* = 2.43
	3	12	14	1			*SD* = 0.73
***Because of Race-Based Discrimination***							
White (*n* = 100)	53.00%	38.00%	7.00%	2.00%			*M* = 1.58
	53	38	7	2			*SD* = 0.71
non-White (*n* = 31)	9.68%	45.16%	38.71%	6.45%			*M* = 2.42
	3	14	12	2			*SD* = 0.76

Note: For each category, the first row contains the percentage and the second row contains the number of responses. Non-White includes Black, Asian, Hispanic, and other non-White individuals collapsed into one group.

To examine differences, we ran a linear regression predicting experiences from the interaction of participant race and question framing. Since we were interested in both main effects and the interaction effect, we opted for effect coding of participant race and simple coding for question framing–this allowed for the intercept to correspond to the mean of all cell means. We ran our initial model with the “Less Respect” framing condition being the reference level for the question framing variable to get a main effect comparing “Less Respect” framing to “Race-Based Discrimination” framing, as well as a main effect comparing “Less Respect” framing to “Any Type of Discrimination” framing. We then ran a follow-up model with “Race-Based Discrimination” as the reference level to get a main effect comparing “Race-Based Discrimination” framing to “Any Type of Discrimination” framing.

**Fig 1:**
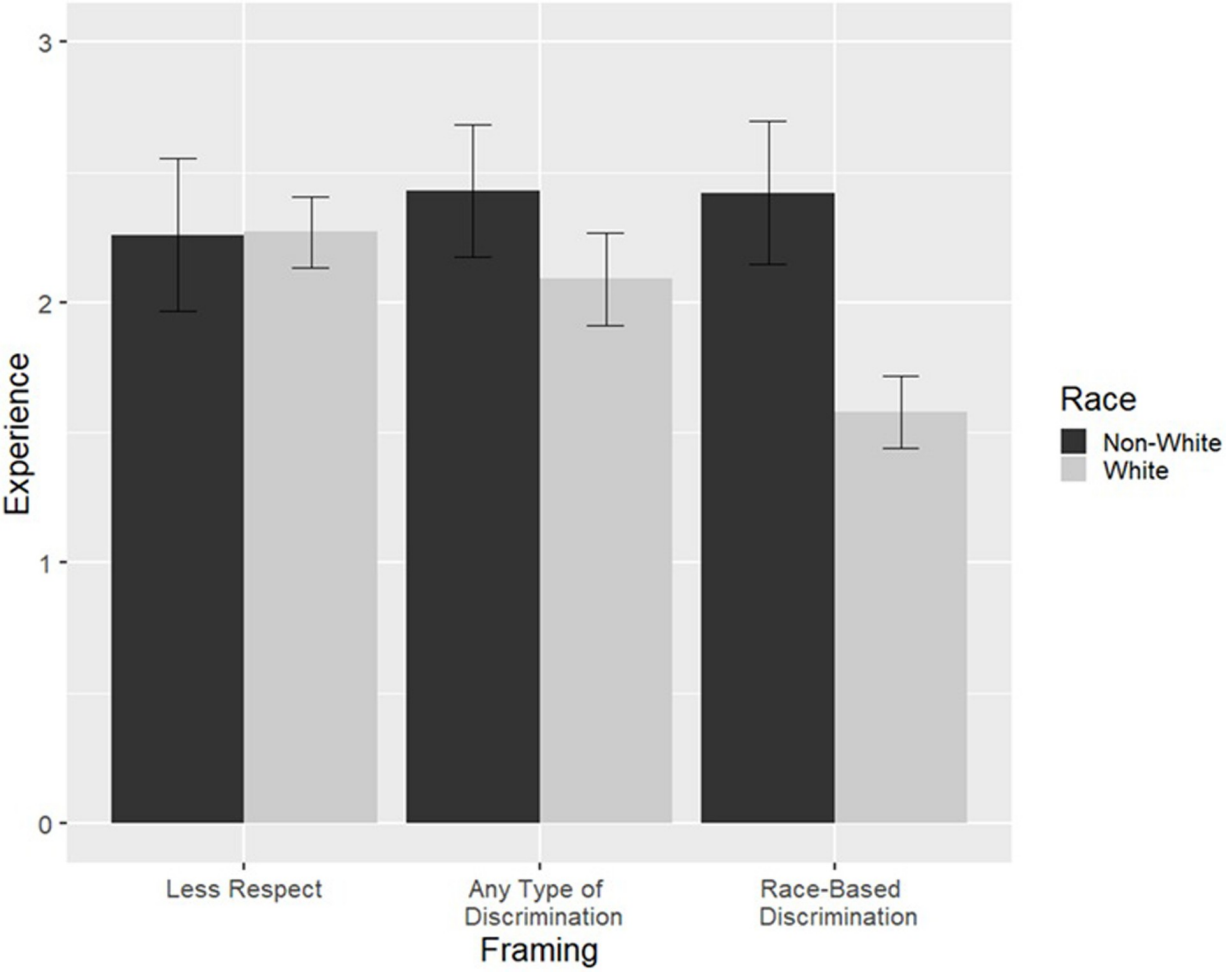
Experiences based on “Less Respect”, “Any Type of Discrimination”, and “Race-Based Discrimination”. Note: non-White includes Black, Asian, Hispanic, and other non-White participants collapsed into one group.

As predicted, we found a main effect of participant race such that regardless of question framing, non-Whites (*M* = 2.37, *SD* = 0.77) reported more experiences than Whites (*M* = 1.98, *SD* = 0.82), *d* = 0.51, *SE* = 0.09, *t*(388) = 4.24, *p* < .001, 95% CI [0.20, 0.57]. These findings were qualified by some important interactions between participant race and question framing: First, non-Whites reported significantly more experiences than Whites in the “Race-Based Discrimination” framing condition, *d* = 1.11, *SE* = 0.16, *t*(388) = 5.32, *p* < .001, 95% CI [0.53, 1.15]. Second, non-Whites reported marginally more experiences than Whites in the “Any Type of Discrimination” framing condition, *d =* 0.44, *SE =* 0.16, *t*(388), *p* = .0350, 95% CI [0.02, 0.66]. Third, we found no differences between non-Whites and Whites in the “Less Respect” framing condition, *d =* 0.02, *SE =* 0.16, *t*(388) = -0.099, *p* = .92, 95% CI [-0.32, 0.29]. Results of a multiple linear regression test indicated that, collectively, there was a significant interaction effect of participant race and question framing, *R*^*2*^ = 0.14, *F*(5, 388) = 12.67, *p* < .001. To control for the familywise error rate in the tests for these interactions, we used the Bonferroni correction procedure (α′ = α/n), thus the significance level for each individual test was set at (.05/3) = .0167. See Fig 1 for a visual representation of the findings.

#### Exploratory analyses

We were interested in looking at possible differences between groups and conducted some exploratory analyses. All following analyses were corrected for multiple comparisons using Tukey’s method for comparing a family of 6 estimates. There was an effect of question framing such that individuals in the “Race-Based Discrimination” framing condition (*M* = 1.78, *SD* = 0.81) reported fewer experiences than individuals in the “Less Respect” framing condition (*M* = 2.27, *SD* = 0.74), *d* = 0.35, *SE* = 0.11, *t*(388) = -2.39, *p* = .0450, 95% CI [-0.53, 0.00], as well as a marginal effect of question framing such that individuals in the “Any Type of Discrimination” framing condition (*M* = 2.17, *SD* = 0.84) reported more experiences than individuals in the “Race-Based Discrimination” framing condition (*M* = 1.78, *SD* = 0.81), *d* = 0.34, *SE* = 0.11, *t*(388) = 2.35, *p* = .0510, 95% CI [-0.00, 0.53]. We did not find an effect between individuals in the “Less Respect” and “Any Type of Discrimination” framing conditions, *d =* 0.00.

Additionally, we looked to see if there were any differences between non-Whites and Whites across framing conditions: Whites in the “Race-Based Discrimination” framing condition reported fewer experiences than non-Whites in the “Any Type of Discrimination” framing condition, *d =* -1.11, *SE* = 0.16, *t*(388) = -5.342, *p* < 0.001, 95% CI [-1.31, -0.40]. Whites in the “Race-Based Discrimination” framing condition reported fewer experiences than non-Whites in the “Less Respect” condition, *d* = -0.88, *SE = 0*.*16*, *t*(388) = -4.299, *p* < 0.001, 95% CI [-0.68, 0.16]. Whites in the “Any Type of Discrimination” framing condition reported more experiences than Whites in the “Race-Based Discrimination” framing condition, *d =* 0.67, *SE* = 0.11, *t*(388) = 0.486, *p* < .001, 95% CI [0.20, 0.83]. Whites in the “Race-based Discrimination” framing condition reported fewer experiences than Whites in the “Less Respect” framing condition, *d = 0*.*90*, *SE = 0*.*11*, *t*(388) = 6.484, *p* < 0.001, 95% CI [0.39, 1.00].

### Discussion

In Study 2, we found evidence to support our hypotheses that participant race and question framing impacts the reporting of experiences. Overall, regardless of whether the question was framed as “Less Respect,” “Any Type of Discrimination,” or “Race-Based Discrimination,” non-White participants reported more experiences than White participants. This effect interacted with question framing. There were no racial differences found in the “Less Respect” framing condition, but racial differences were found in the “Race-Based Discrimination” framing condition, such that non-White participants reported more experiences than White participants. In Sum, a broad framing of “Less Respect” led to a minimization of reported racial differences, whereas more specific framings (e.g., “Race-Based Discrimination”) led to a more pronounced difference between non-White participants and White participants. This study was limited such that non-native English speakers were excluded due to our inclusion criteria, which likely led to conservative estimates of prevalence of experiences and framing effects. We note that past research has found that non-English and non-native English speakers are exposed to higher rates of discriminatory treatment (e.g., [24,25]). Additionally, we were not able to examine racial differences within non-White groups.

## General discussion

Our studies provide evidence that the “possibly exaggerated claims that discrimination is a prevalent feature of contemporary life in the United States” [1] may be less exaggerated than Boutwell et al. report. In sum, our findings present a strikingly different picture on the prevalence of discrimination in the United States and provide insight as to why the prevalence rates of Boutwell and colleagues were so different than that of previous research, as well as Study 1.

In Study 1, we found a much higher prevalence rate of discrimination than Boutwell et al. report across all racial groups, regardless of how we analyzed the data. For example, Boutwell et al. found that 31.88% of Blacks experienced discrimination, whereas we found a range of 69.45% and 73.62% depending on how we dichotomized experiences. Replicating previous research (e.g., [26–28]), we found that non-Whites in the United States experience more discrimination than their White counterparts.

In Study 2, we examined whether manipulating the framing of the question used by Boutwell et al. on experiences of less respect and courtesy would lead to different responses by framing condition and participant race. Regardless of framing condition, non-White participants reported more experiences than White participants. In the broad “Less Respect” framing condition, there were no differences between non-White and White experiences. However, in the framing condition where participants were asked about experiences caused by “Race-Based Discrimination,” non-Whites reported more experiences than Whites. These findings highlight the importance of considering question framing when asking participations about their discrimination experiences.

We note that Boutwell and colleagues [1] believed that “much caution is necessary when interpreting” their findings and that “the conclusion that [their] results suggest that the problem of discrimination in the US is, to any great extent, remedied and in need of further scrutiny or improvement” should be avoided. We agree with them, and it is unfortunate that many readers of their work did not heed their call. Further, although other researchers (see Everett, Onge, & Mollborn [29]) have used the Add Health Wave IV item as an index of discrimination (in their case to examine minority status and the item’s relationship with mental health outcomes), our findings suggest that attention to the wording in that work may yield different theoretical conclusions.

The current results are consistent with previous research on the prevalence of discrimination experiences in the United States. Still, there is evidence that discrimination prevalence rates may be even higher than what is shown in our results. Age, socioeconomic status, and legal status have been found to influence how likely one is to experience and/or report discriminatory treatment based on race or ethnicity [30,16]. Research by Crosby [31] suggests that people are prone to minimize personal experiences of discrimination because of the difficulty of inferring discrimination from individual cases and because of the discomfort in confronting one’s own victimization. Similarly, individuals perceive a higher level of discrimination directed at the group they belong to as a whole than themselves as individuals [32]. Indeed, studies have shown that individuals do not label their own experiences as discrimination, even if their experiences fit the definition used by researchers and policymakers [33]. Conversely, individuals may perceive and report experiencing discrimination, even if researchers and policymakers do not categorize their experiences as discrimination.

A rich body of literature suggests that discrimination exists and has real outcomes. Robust discrepancies between Whites and non-Whites are found in hiring [34–36], healthcare access [37], health outcomes [38–41], housing [42], lending [43], education, [44], and prosecution and sentencing [45,46]. We believe that these are the indices that best capture how discrimination should be thought of because they are the tangible manifestations of beliefs, attitudes, and feelings. These discrepancies should be considered and taken into account when examining or interpreting individual experiences of discrimination.
